# Supplementary material for: Organized Breast and Cervical Cancer Screening: Attendance and Determinants in Southern Italy
Source: Cancers (Basel). 2021 Mar 30;13(7):1578. doi: 10.3390/cancers13071578 (PMC8036794; doi:10.3390/cancers13071578)
Supplement: Supplementary file 1 [file cancers-13-01578-s001.pdf]

cancers MDPI

S1 Supplementary File
Questionnaire

Section A. Socio-demographics

A1. Age ____ **A2.** Nationality ____

A3. What is your education level? ____

A4. What is your current occupation? ____

A5. What is your marital status? □ Married □ Unmarried □ Other, (specify _____________________)

A6. How many children do you have? ____

Section B. Anamnestic Information

B1. Do you currently suffer from one or more of the following diseases?

□ Hepatitis B □ Hepatitis C □ HIV □ Diabetes □ Asthma □ Allergies □ Heart disease □ COPD

□ Hypertension □ Cancer □ Arthrosis □ Depression □ Anxiety □ Other, (specify ____________)

B2. Have you smoked at least 100 cigarettes in all your life? □ No (**go to question B5**) □ Yes

B3. Do you currently smoke cigarettes? □ No, I stopped smoking (**go to question B5**) □ Yes

B4. How many cigarettes have you smoked per day in the last week?

□ 1 □ 2-5 □ 6-10 □ 11-20 □ More than 20

B5. Do you drink alcohol? □ No (**go to question B7**) □ Rarely □ Sometimes □ Often □ Every day

B6. Do you intake more than 1 glass of wine or 330 ml of beer in a day? □ No □ Yes

B7. Have you practiced physical activity in the last month (e.g. football, basketball, swimming, running, gym, volleyball, tennis)? □ No □ Yes

B8. How would you classify your current health status? (1 poor; 10 excellent)___

B9. Have you or anyone in your family (e.g. parents, brothers, sisters) ever had cancer?

□ No □ Yes (who _____________________________ what kind of cancer _________________)

Section C. Knowledge

C1. On your opinion, what are the most frequent types of cancer in women? **(more than one answer allowed)**

□ Bowel cancer □ Lung cancer □ Breast cancer □ Cervical cancer □ Oral cancer

□ Anus cancer □ Thyroid cancer □ Other, (specify___________________________________)

C2. Screening tests can detect cancer at an early stage (e.g. mammography, Pap smear test, etc.). Have you ever heard about them?

□ No □ Yes, by whom? □ Physicians □ Friends □ Internet □ Other, (specify_______________)

C3. On your opinion, which one of the following types of cancer can be detected early? **(more than one answer allowed)**

□ No one □ Bowel cancer □ Lung cancer □ Breast cancer □ Anus cancer □ Cervical cancer

□ Prostate cancer □ Oral cancer □ Other, (specify___________________________________)

Section D. Attitudes

D1. How much are you worried about developing cancer? (1 not at all worried; 10 very worried) _____

D2. How much are screening tests useful to prevent cancer? (1 not at all useful; 10 very useful) ______

Section E. Behaviours

E1. Have you been examined by a physician in the last 12 months?

□ No **(go to question E3)** □ Yes, by a general practitioner □ Yes, by a specialist (e.g. gynecologist, urologist) □ Yes, by others (specify____)

E2. Why have you consulted a physician in the last 12 months? **(more than one answer allowed)**

□ Periodic check-up □ Preventive purposes (e.g. vaccination) □ Other, (specify__________________)

E3. Have you ever undergone any of the following tests? **(answer to each of the following)**

E3.1. Mammography uses X-ray to detect breast cancer and is recommended for women from 45 to 69 years old every 2 years. Have you ever undergone it?

□ **Yes for control** (specify when was the last time you underwent the test ________)

Why? (Specify the reason)

□ I received the invitation and appointment by the Local Health Unit

□ It was recommended by Primary Care Physician

□ It was recommended by a healthcare practitioner (e.g. gynecologist, breast specialist, oncologist)

□ It was my own initiative

□ **Yes, because of health problems**

□ **No:** □ I do not receive any invitation by Local Health Unit □ I did not know it was free

□ I have not been advised □ Too long waiting list □ I am not in the target age range

□ I am afraid of pain □ I am afraid of discovering the disease

□ Lack of time □ I do not have any health problem

□ Other, (specify____)

□ **I do not remember**

E3.2. Pap smear test collects cells from uterus to detect cervical cancer and is recommended for women aged 25-64 every 3 years. Have you ever undergone it?

□ **Yes for control** (specify when was the last time you underwent the test ________)

Why? (Specify the reason)

□ I received the invitation and appointment by the Local Health Unit

□ It was recommended by Primary Care Physician

□ It was recommended by a healthcare practitioner (e.g. gynecologist, oncologist)

□ It was my own initiative

□ **Yes, because of health problems**

□ **No:** □ I do not receive any invitation by Local Health Unit □ I did not know it was free

□ I have not been advised □ Too long waiting list □ I am not in the target age range

□ I am afraid of pain □ I am afraid of discovering the disease

□ Lack of time □ I do not have any health problem

□ Other, (specify____)

□ **I do not remember**

E3.3. DNA HPV-test collects cells from the uterus to detect the Papilloma Virus (HPV) and is recommended for women aged 30-35 to 64 every 5 years. Have you ever undergone it?

□ **Yes for control** (specify when was the last time you underwent the test _______)

Why? (Specify the reason)

□ I received the invitation and appointment by the Local Health Unit

□ It was recommended by Primary Care Physician

□ It was recommended by a healthcare practitioner (e.g. gynecologist, oncologist)

□ It was my own initiative

□ **Yes, because of health problems**

□ **No:** □ I do not receive any invitation by Local Health Unit □ I did not know it was free

□ I have not been advised □ Too long waiting list □ I am not in the target age range

□ I am afraid of pain □ I am afraid of discovering the disease

□ Lack of time □ I do not have any health problem

□ Other, (specify____)

□ **I do not remember**

E4. If you have not undergone cancer screening because you are not in the right target age range, would you undergo it if you were?

□ No, I would not (because_______________________________________)

□ Yes, I would undergo mammography

□ Yes, I would undergo DNA HPV test

□ Yes, I would undergo Pap smear test

□ Yes, I would undergo FOBT

E5. Have you received HPV vaccination? □ No □ Yes **(go to question E7)**

E6. Have you ever taken part to cancer prevention activities (training courses, information material, etc.)? □ No

□ Yes (specify activity ________________________ and organizers____________________)

F. <u>INFORMATION ABOUT CANCER PREVENTION</u>

F1. What are your sources of information about cancer prevention? **(more than one answer allowed)**

□ None □ TV, newspapers □ Physicians □ Friends, relatives □ Internet □ Other, (specify___________)

F2. Do you need more information concerning behaviors to prevent cancer?

□ No, why? ____

□ Yes, from: □ TV, newspapers □ Physicians □ Friends, relatives □ Internet □ Other, (specify______)

S2 Supplementary File

Variables included in the logistic regression models with related categories

Model 1. Accurate knowledge (about the most frequent cancers in females and cancers that could be detected early).

Independent variables	Code
Age, in years (categorical)	28-44=3
	45-49=2
	50-67=1
Employment status	No=0
	Yes=1
Education level	Undergraduate=0
	Graduate=1
Marital status	Unmarried=0
	Married=1
Personal history of chronic diseases	No=0
	Yes=1
Family history of breast cancer	No=0
	Yes=1
Having been informed about cancer prevention by physicians	No=0
	Yes=1

Model 2. Attendance to Pap-smear within organized programs in the previous three years.
Model 3. Overall attendance to Pap-smear in the previous three years.

**Independent variables**	**Code**
Age, in years	28-44=3
	45-49=2
	50-67=1
Employment status	No=0
	Yes=1
Education level	Undergraduate=0
	Graduate=1
Personal or family history of cancer	No=0
	Yes=1
Family history of breast cancer	No=0
	Yes=1
Current smokers	No=0
	Yes=1
Alcohol consumption	No=0
	Yes=1
Physical activity	No=0
	Yes=1
Knowledge that some cancers can be detected early	No=0
	Yes=1
Knowledge of which cancers can be detected early	No=0
	Yes=1
Perception of personal health status	Unsatisfactory (1-9) =0
	Satisfactory (10) =1
Perceived risk of developing cancer, continuous	Range 1-10
Perceived effectiveness of screening tests, continuous	Range 1-10
Having visited a physician in the previous 12 months	No=0
	Yes=1
Having been informed about cancer prevention by physicians	No=0
	Yes=1
Need of additional information about cancer prevention from physicians	No=0<br>Yes=1

Model 4. Attendance to mammography within organized programs in the previous two years.
Model 5. Overall attendance to mammography in the previous two years.

**Independent variables**	**Code**
Age, in years	28-44=1
	50-67=0
Employment status	No=0
	Yes=1
Education level	Undergraduate=0
	Graduate=1
Personal or family history of cancer	No=0
	Yes=1
Family history of breast cancer	No=0
	Yes=1
Current smokers	No=0
	Yes=1
Alcohol consumption	No=0
	Yes=1
Physical activity	No=0
	Yes=1
Knowledge that some cancers can be detected early	No=0
	Yes=1
Knowledge of which cancers can be detected early	No=0
	Yes=1
Perception of personal health status	Unsatisfactory (1-9) =0
	Satisfactory (10) =1
Perceived risk of developing cancer, continuous	Range 1-10
Perceived effectiveness of screening tests, continuous	Range 1-10
Having visited a physician in the previous 12 months	No=0
	Yes=1
Having been informed about cancer prevention by physicians	No=0
	Yes=1
Need of additional information about cancer prevention from physicians	No=0
	Yes=1
